# A Novel Cardenolide Glycoside Isolated from *Xysmalobium undulatum* Reduces Levels of the Alzheimer’s Disease-Associated β-Amyloid Peptides Aβ42 In Vitro

**DOI:** 10.3390/ph14080743

**Published:** 2021-07-29

**Authors:** Anuradha Thakur, Phanankosi Moyo, Carl Johan van der Westhuizen, Hyun Ok Yang, Vinesh Maharaj

**Affiliations:** 1 Department of Chemistry, University of Pretoria, Pretoria 0028, South Africa; apti.0121@gmail.com; 2 Department of Biochemistry, Genetics and Microbiology, Institute for Sustainable Malaria Control, University of Pretoria, Hatfield, Pretoria 0028, South Africa; u13386842@tuks.co.za; 3 Future Production: Chemicals, Council for Scientific and Industrial Research (CSIR), Meiring Naudé Road, Pretoria 0001, South Africa; JvdWesthuizen1@csir.co.za; 4 Natural Products Research Centre, Korea Institute of Science and Technology, Gangneung 25451, Gangwon-Do, Korea; 5 Department of Integrative Biological Sciences and Industry, Sejong University, Seoul 05006, Korea

**Keywords:** *Xysmalobium undulatum*, Aβ42 reduction, nuclear magnetic resonance (NMR), acetylated glycosydated crotoxigenin, xysmalogenin-3, β-d-glucopyranoside, Alzheimer, natural products

## Abstract

Elevated levels of the amylo β-proteins (Aβ), particularly Aβ42, are associated with a high risk of Alzheimer’s disease (AD). The Aβ proteins are produced from cellular processing of the amyloid precursor proteins (APPs). To identify natural products that block the formation of Aβ-proteins from APPs, we previously screened a library of plant extracts and identified *Xysmalobium undulaum* (Apocynaceae) as a potential plant for further research. Here, we provide a report on the isolation and identification of the active principles from the plant species using a bioassay-guided fractionation. Fractions and resulting pure compounds from the purification process of the extract of *X. undulatum* were screened in vitro against APPs transfected HeLa cell lines. Three compounds, acetylated glycosydated crotoxogenin (**1**), xysmalogenin-3, β-d-glucopyranoside (**2**), and crotoxigenin 3-*O*-glucopyranoside (**3**), were subsequently isolated and their structures elucidated using NMR and mass spectrometry. Compound **1**, a novel cardenolide, and **2** significantly decreased the Aβ42 levels in a dose-dependent manner while compound **3** was inactive. In silico investigations identified the AD’s β-secretase enzyme, BACE1, as a potential target for these compounds with the glycoside moiety being of significance in binding to the enzyme active site. Our study provides the first report of a novel cardenolide and the potential of cardenolides as chemical scaffolds for developing AD treatment drugs.

## 1. Introduction

Alzheimer’s disease (AD) is a progressive debilitating neurodegenerative disorder. It is the most prevalent cause of dementia, a clinical syndrome that currently affects 50 million people worldwide [1]. There were only four FDA approved AD treatment drugs, with the last candidate brought into the market in 2003 [2]. After a long wait, aducanumab, was approved by the FDA using the accelerated approval pathway, for AD treatment in June of 2021. Aducanumab is an amyloid beta (Aβ)-directed antibody indicated to treat AD [3].

The mechanism of action for three of these four low molecular agents, namely donepezil, galantamine, and rivastigmine, is through the inhibition of acetylcholinesterase whilst one, memantine, is an *N*-Methyl-d-aspartate receptor antagonist. Clinical benefit of these drugs is unfortunately only modest with data from clinical trials showing no significant difference in decline of cognitive function in patients treated with these drugs compared with those taking placebo [4]. This lack of effective AD treatment regimens, combined with a high attrition rate of clinical candidate drugs [2], strongly underlies the need to discover and develop novel drugs, ideally with a unique mechanism of action, for the treatment of this disease. Against this background, in our quest to discover and develop new drugs for the treatment of AD, we previously screened a library of 33 plant extracts for their ability to reduce the amyloid β-proteins, specifically in vitro inhibition of Aβ42 (a well characterised druggable AD target [5]), leading to the identification of *Xysmalobium undulatum* (Apocynaceae) as a potent hit plant species [6]. This plant is extensively harvested for its medicinal use in South Africa [7]. Traditionally, extracts of the plant are used for the treatment of diarrhea, dysentery, dysmenorrhea, stomach cramps, intestinal problems, wounds, and indigestion [8]. The root extract of *X. undulatum* is marketed as a herbal drug, under the name “Uzara”, for the treatment of acute diarrhea [9]. Phytochemical investigations have identified cardenolide glycosides as the main class of compounds in *X. undulatum*. This plant is a prolific inhibitor of the enzyme acetylcholinesterase (IC_50_ = 0.5 ng/mL) [10] which makes it more enticing to interrogate in search of pan-active AD treatment drugs.

Using a bioassay-guided approach, we previously isolated two compounds which potently inhibited formation of Aβ42 in vitro [6]. As a follow up to our prior investigation, herein, we provide a first report on the isolation of three more compounds from *X. undulatum*, including one which is novel, with two of these compounds significantly reducing the Aβ42 protein levels in vitro and hence marking them as potential chemical scaffolds for the development of new AD drugs.

## 2. Results

### 2.1. Isolation of Compounds from X. Undulatum Using Bioassay-Guided Strategy

**Figure 1:**
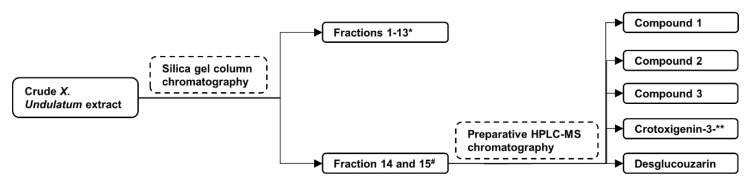
Illustration of the bioassay guided isolation of compounds **1**, **2,** and **3** from *X. undulatum* crude extract. These compounds where isolated from the same fractions (14 and 15) from which the cardenolides ** crotoxigenin-3-*O*-β-digitalopyranosyl-(1-4)-*O*-β-digitoxopyanoside and desglucouzarin where isolated from [6]. * Fractions 1-13 previously demonstrated poor activity (<20% inhibition of Aβ42 formation) while ^#^ fractions 14 and 15 both showed good activity (>70% inhibition of Aβ42 formation) at 50 μg/mL.

From a starting material of 200 g of dried leaves, 17 g of dry crude extract was obtained (8.5% yield) after extraction with dichloromethane and methanol (DCM: MeOH) (1:1) and evaporation of the solvent. An amount of 15 g of the crude extract was subjected to purification by silica gel column chromatography resulting in the generation of 15 pooled fractions (based on TLC analysis) which was consistent with the previous study [6]. Two of the fractions (14 and 15), with phytochemical profile matching that of the two previously identified potent fractions [6], were further subjected to additional purification using preparative HPLC-MS resulting in the isolation of three compounds (**1**, **2,** and **3**) which were then subjected to structure elucidation (Figure 1).

Compound **1** (4 mg) was isolated as a white amorphous powder. It had a molecular formula of C_31_H_44_O_11_ (calculated mass 592.2883) (Figure S1), as deduced from its pseudomolecular precursor ion at *m*/*z* 637.2851[M-HCOO] based on the mass spectrum. This data confirmed the presence of cardenolide glycoside. The formula was further confirmed by the number of protons in ^1^H NMR and of carbon atoms in the ^13^C NMR spectrum (Figures S2 and S3).

The ^1^H NMR spectrum (MeOD-*d4*, 400 MHz) of the compound showed the H-21 protons at δ 5.03 (1 H, dd) and δ 4.96 (1 H, dd) and an olefinic proton at δ 5.92 (1 H, s, H-22) as part of the lactone ring which indicated the characteristic feature of the cardenolide system. The other prominent signal indicated the presence of one up field methyl proton at δ 0.98 (3 H, s, H-18). The spectrum also indicated one downfield aldehydic proton at δ 9.57 (1 H, s, H-19) and overall pattern of the ^1^H NMR indicated that the compound was a cardenolide with one sugar unit as an anomeric proton signal was observed at δ 4.40 (1 H, d, H-1′). The chemical shift at δ 2.08 (3 H, s, 5′-OCOCH_3_) confirmed the presence of an acetate group, in all likelihood attached to the sugar moiety.

The ^13^C NMR spectrum had a total of 31 signals and the assignments were done together with the DEPT 135 spectra (Figure S4). The methylene signal at δ 73.9 was assigned to C-21, δ 116.4 assigned to C-22 olefinic carbon, δ 176.9 assigned to the C-23 carbonyl group, and δ 177.8 assigned to the quaternary C-20, all of which confirmed the presence of the butenolide ring. Two prominent signals at δ 14.8 assigned to C-18 and at δ 206.5 assigned to C-19 indicated the position of methyl carbon and carbonyl carbon, respectively. The β hydroxyl group at C-14 was confirmed by the downfield chemical shift at δ 84.5. One anomeric carbon was revealed at δ 101.3 which was assigned to C-1′. The carbon atom at δ 171.2 (5′-OCOCH_3_) revealed the presence of the acetate group and the carbon signal at δ 19.3 represented the position of the methyl group.

The HSQC and COSY correlations (Figures S5 and S6) led to the assignment of the proton and carbon signals. The chemical shifts for ^1^H and ^13^C are shown in Table 1. The strong HMBC correlation (Figure S7) between the proton at δ 2.80 (H-17) with C-21 (δ 73.9) and C-22 (δ 116.4) confirmed that the *α,β*-unsaturated *γ*-lactone was connected at C-17 (δ 50.4). The proton at δ 9.57 (H-19) is correlated to C-9 (δ 35.1), which confirmed the position of the aldehydic proton. HMBC correlations between C-5-CH_3_ with C-5-OCO confirmed the presence of the acetate group. HMBC correlations also established the linkages of the glycosidic bonds and the point of attachment of the saccharide chain to the aglycone based on the correlation between δ 4.40 (H-1) to δ 77.8 (C-3). The proton at δ 3.10 (H-2) correlating with δ 101.3 (C-1) and δ 73.7 (C-4); and proton δ 3.30 (H-4) correlating with δ 76.48 (C-5) and δ 63.40 (C-6) confirmed a single sugar moiety.

**Figure 2:**
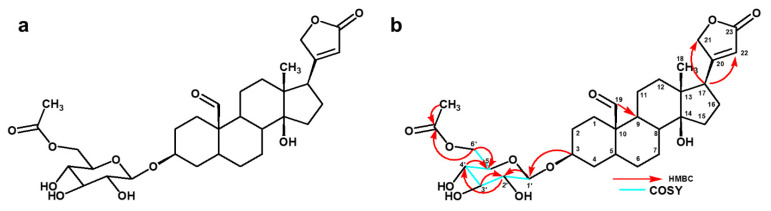
Cardenolide glycoside isolated from *X. undulatum*, (**a**) acetylated glycosydated crotoxigenin (**1**), and (**b**) its key HMBC and COSY correlations.

The large coupling constant of the anomeric proton at (δ 4.40, 7.57 Hz) indicated the β- orientation of the glycoside moiety [11,12]. There was a COSY correlation between the anomeric proton at δ 4.4 (H-1′) and the proton at δ 3.17 (H-2′) while another correlation was observed between the proton at δ 3.17 (H-2′) and the proton at δ 3.36 (H-3′). The proton at δ 3.30 (H-4′) showed a COSY correlation with the proton at δ 3.47 (H-5′). The proton at δ 3.47 (H-5′) coupled with the two methylene protons at δ 4.20 (C6′a) and δ 4.30 (C6′b) to give a multiplet. These methylene protons also coupled which each other, hence the two sets of doublets at δ 4.20 (*dd*, 11.6 Hz, 5.14 Hz, H-6′a) and δ 4.30 (*dd*, 11.6 Hz, 1.84 Hz, H-6′). All these correlations are similar to that of the glucopyranoside attached to the aglycone moiety which is similar to compound 3 except the attachment of the acetoxy group at C6′ which makes it novel and it is the first time it is reported at this position in the sugar moieties present in cardenolide glycosides. The HMBC and COSY correlations are shown in Figure 2. Taking all this spectral data into account, compound **1** was subsequently identified as a novel acetylated glycosydated crotoxigenin (Figure 2).

Compound **2** was identified as a known compound, xysmalogenin-3, β-d-glucopyranoside based on the NMR data, mass spectral analysis, and previous published studies [10]. However, the exact and complete NMR data for this compound were not previously published. It was isolated as a white solid weighing 3.5 mg and had a molecular formula of C_29_H_42_O_9_ (calculated mass 534.2828) (Figure S8), as deduced from its pseudomolecular precursor ion at *m*/*z* 579.2822 [M-HCOO]^−^ based on the mass spectrum. These data confirmed the presence of cardenolide glycoside. The formula was further confirmed by the number of protons in ^1^H NMR and of carbon atoms in the ^13^C NMR spectrum (Figures S9 and S10).

The ^1^H NMR spectrum (MeOD-*d4*, 400 MHz) of the compound showed the H-21 protons at δ 5.02 (1 H, dd) and δ 4.92 (1 H, dd) and an olefinic proton at δ 5.91 (1 H, s, H-22) as a part of the lactone ring which indicated the characteristic feature of the cardenolide system. The spectrum showed the presence of one more double bond at δ 5.44 (1 H, s, H-6). The other prominent signals indicated one high field methyl proton at δ 0.92 (1 H, s, H-18), and another high field proton at δ 1.03 (1 H, s, H-19). The spectrum indicated the cardenolide with one sugar unit, with a signal for one anomeric proton at δ 4.38 (1 H, dd, H-1), where the large coupling constant (7.68 Hz) confirmed the β-coupling of the sugar unit to the aglycone.

The ^13^C NMR spectrum had a total of 31 signals and the assignments were done together with the DEPT 135 spectra (Figure S11). The methylene signal at δ 75.2 was assigned to C-21, δ 117.8 assigned to C-22 olefinic carbon, δ 178.2 assigned to the C-23 carbonyl group, and δ 177.1 assigned to the quaternary C-20, all of which confirmed the presence of butenolide ring. Two prominent signals at δ 16.1 assigned to C-18 and at δ 19.8 assigned to C-19 indicated the position of two methyl carbons. The β hydroxyl group at C-14 was confirmed by downfield chemical shift at δ 86.3. One anomeric carbon revealed at δ 102.3 was assigned to C-1′.

The proton and carbon signals are assigned based on the HSQC (Figure S12) and COSY correlations (Figure S13). The chemical shifts for ^1^H and ^13^C are shown in Table 2. The α, β- unsaturated γ-lactone was determined to be connected at C-17 through the strong HMBC (Figure S14) correlations between protons at δ 2.86 (H-17) to δ 177.1 (C-21), δ 117.8 (C-22), and δ 178.8 (C-23). The glycosidic linkage and the point of attachment to the aglycone-genin moiety was established by HMBC correlations from δ 4.38 (H-1) to δ 79.6 (C-3). HMBC correlations between δ 3.14 (H-2) with δ 102.3 (C-1) and δ 78.0 (C-3); and (δ 3.84) H-6a with δ 77.7 (C-5) and δ 71.5 (C-4), confirmed the sugar unit.

**Figure 3:**
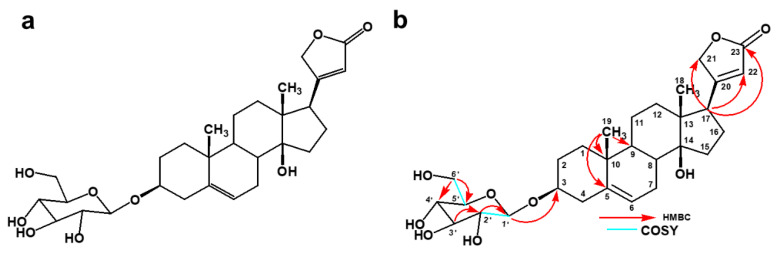
Cardenolide glycoside isolated from *X. undulatum*, (**a**) xysmalogenin-3, β-d-glucopyranoside (**2**), and (**b**) its key HMBC and COSY correlations.

A large coupling constant H1′/H2′ (δ 4.38, 7.68 Hz) indicated a diaxial relationship for anomeric proton H1′ and proton H2′. This large coupling constant also indicated the β-pyranose form for glycoside by verifying the β-orientation of glycoside. COSY correlations were also observed between δ 5.44 (H-6) and δ 2.26 (H-7). The HMBC and COSY correlations are shown in Figure 3.

**Figure 4:**
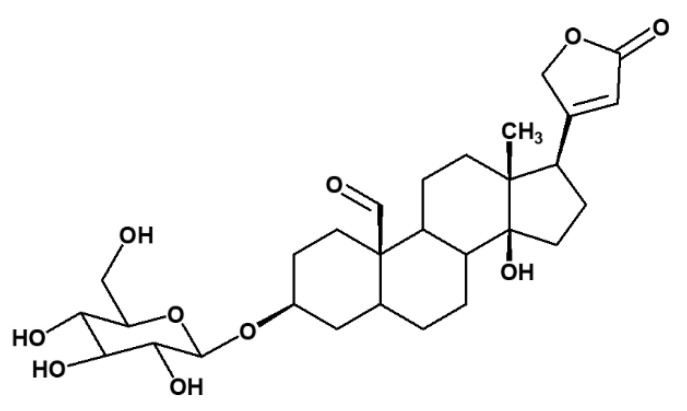
Cardenolide glycoside crotoxigenin 3-*O*-glucopyranoside (**3**), isolated from *X. undulatum*.

Compound **3** was identified as a known compound crotoxigenin 3-*O*-glucopyranoside based on the NMR data, mass spectral analysis, and previous published data (Figure 4) [13]. It was isolated as a white crystalline solid (5 mg). It had a molecular formula of C_29_H_42_O_10_ (calculated mass 552.2934) (Figure S15), as deduced from its pseudomolecular precursor ion at *m*/*z* 595.2758 [M-HCOO]^−^ based on the mass spectrum. These data confirmed the presence of cardenolide glycoside. The ^1^H and ^13^C NMR data of compound **3** compared favourably with the NMR data based on the literature data for the compound (Table S1) [13].

### 2.2. In Vitro Inhibition of Aβ42 Production by Compounds Isolated from X. Undulatum

**Figure 5:**
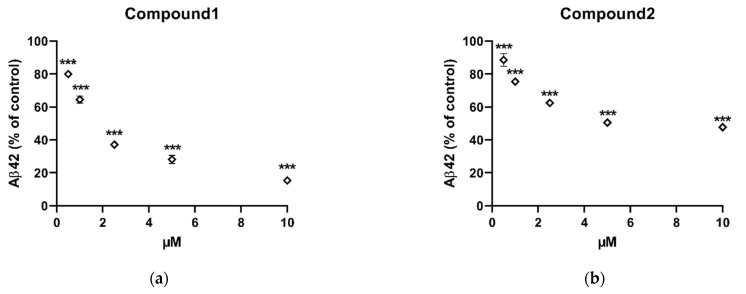
Changes in the level of Aβ42, following exposure to (**a**) compound **1** and (**b**) compound **2** in vitro. Cells were incubated with indicated concentrations of compounds for 8 h. Statistically significant differences in % of inhibition values are indicated (*** *p* < 0.001, one-way ANOVA). Data are presented as mean ± SEM (*n* = 4). DMSO served as negative control.

Following the isolation and identification of the three compounds, the next step was to subject them to in vitro pharmacological profiling using the Aβ peptide assay primarily to interrogate their ability to inhibit Aβ42 production in HeLa cells stably transfected with APPsw. Dimethyl sulfoxide (DMSO), as the solvent, served as a negative control showing no activity in reducing Aβ42 production in vitro. Of the three compounds, compound **1** was the most potent (*n* = 4, *p* < 0.001, one-way ANOVA) decreasing the levels of Aβ42 by 20.05 ± 1.6%, 35.53 ± 2.1%, 62.83 ± 1.6%, 71.85 ± 2.4%, and 84.65 ± 0.1% in a dose dependent manner at concentrations of 0.5, 1, 2.5, 5, and 10 μM, respectively (Figure 5a). Compound **2** was the second most active significantly (*n* = 4, *p* < 0.01, one-way ANOVA) decreasing the Aβ42 levels by 6.91 ± 3.8%, 24.7 ± 0.0%, 37.6 ± 0.1%, 49.5 ± 0.0%, and 52.3 ± 1.9% in a dose dependent manner at 0.5, 1, 2.5, 5, and 10 μM, respectively (Figure 5b). Compound **3** was inactive at the highest test concentration of 10 μM.

### 2.3. In Vitro Inhibition of Aβ40 and Sappβ-Sw Production by Compound **1**

**Figure 6:**
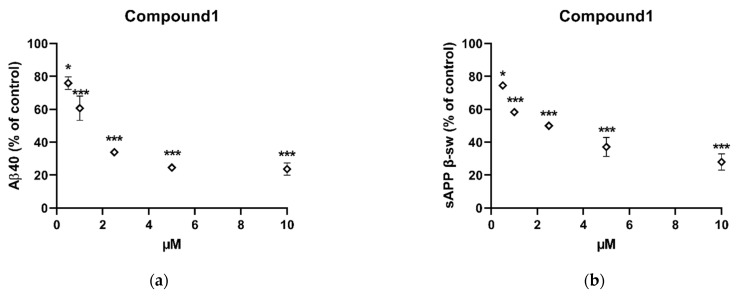
Changes in the level of (**a**) Aβ40 and (**b**) sAPPβ-sw following exposure to compound **1** in vitro. Cells were incubated with indicated concentrations of the compound for 8 h. The levels of Aβ40 and sAPPβ-sw were measured from the conditioned media by using ELISA. Statistically significant differences in % of inhibition values are indicated (* *p* < 0.05 and *** *p* < 0.001, one-way ANOVA). Data are presented as mean ± SEM (*n* = 4). DMSO served as negative control.

Having emerged as the most active compound, compound **1** was prioritized for further investigation by measuring the secreted levels of APP proteolytic products (Aβ40 and sAPPβ-sw) from the conditioned media using specific ELISA kits. The level of Aβ40 was substantially decreased in a dose-dependent manner by 24.07 ± 3.9%, 39.30 ± 7.4%, 66.12 ± 2.0%, 75.39 ± 0.9%, and 76.30 ± 3.8% at 0.5, 1, 2.5, 5, and 10 μM, respectively (Figure 6a). Similarly, the level of sAPPβ-sw was also notably decreased in a dose-dependent manner by 25.57 ± 0.5%, 41.68 ± 2.0%, 49.99 ± 0.9%, 62.91 ± 5.8%, and 72.03 ± 4.9% at 0.5, 1, 2.5, 5, and 10 μM, respectively (Figure 6b). These results suggest that this compound decreased Aβ42 production at non-toxic concentrations by decreasing amyloidogenic processing of APP which is proven to exhibit the neuroprotective properties and enhance memory [14].

### 2.4. Binding Pose Analysis of Compound **1** in Β-Site Amyloid Precursor Protein Cleaving Enzyme 1 Active Site

To investigate the mechanism of action of the isolated compounds, compounds (ligands) **1**–**3** were docked into an ensemble of receptors to predict the binding pose. Using an ensemble of receptor conformations for docking improves the probability that the correct binding pose is obtained. However, while the binding poses from *Glide* looked reasonable based on visual inspection, the docking scores obtained were poor.

**Figure 7:**
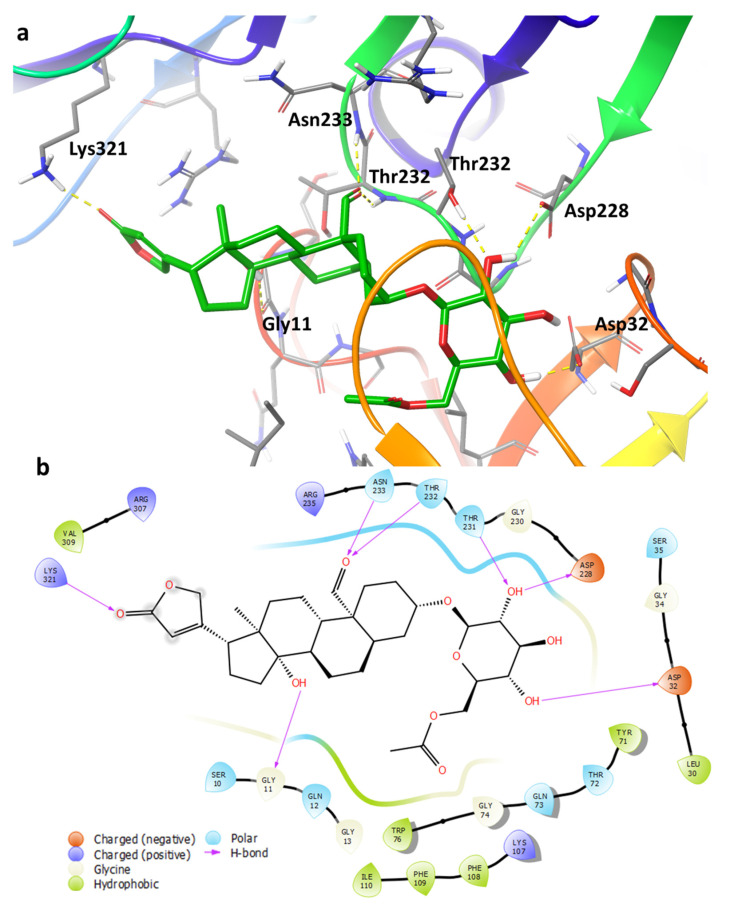
(**a**) Predicted binding pose from induced-fit docking for compound (ligand **1**), shown in green, bound to BACE1. This binding pose, which is similar to the poses predicted for ligands **2** and **3**, forms several hydrogen-bonds with the surrounding residues shown in yellow dashed lines. Only selected residues are shown for clarity. (**b**) 2D ligand interaction diagram for the binding pose of **1**.

Induced fit docking (IFD) was subsequently used to identify alternative binding poses. The majority of the poses predicted were related to one another. Figure 7 shows the predicted binding pose of the novel ligand **1** with β-site amyloid precursor protein cleaving enzyme 1 (BACE1). The predicted binding poses of ligands **2** and **3** are provided in the (Figures S16 and S17). The following hydrogen bonds are noted: the glycoside hydroxyl groups form interactions with the amino acid residues Asp32, Asp228, and Thr231; the aldehyde forms hydrogen bonds with the backbone of Thr232 and Asn233; and the hydroxyl of the steroid moiety interacts with the backbone of Gly11. Furthermore, the butanolide ring forms interaction with Lys321, however, due to this moiety being exposed to the solvent it is unlikely to be a critical interaction.

## 3. Discussion

As the number of people afflicted with AD continues to rise globally, new effective therapies are needed to turn the tide. Natural products are an inspired staring source to explore as they have served as a mainstay reservoir of chemical scaffolds that have been used to develop numerous drugs. Close to 50% of pharmaceutical drugs are either made from natural products or structurally inspired by natural products [15]. Alzheimer’s disease treatment regimens have not been an exception to this. Of the four drugs currently available for its treatment, two of them, galantamine and rivastigmine, are of natural origin. The alkaloid galantamine was originally isolated from bulbs of *Galanthus nivalis* (Amaryllidaceae) [16], while rivastigmine is a semi-synthetic derivative of the natural compound physostigmine, an alkaloid that occurs naturally in the plant species *Physostigma venenosum* (Fabaceae) [17]. Against this background, our group has been motivated to probe unique natural products, of plant origin found in South Africa, in search of novel AD treatment drugs. Using the classical bioassay-guided approach, this endeavour has led to the discovery of a novel potent cardenolide from *X. undulatum*.

**Figure 8:**
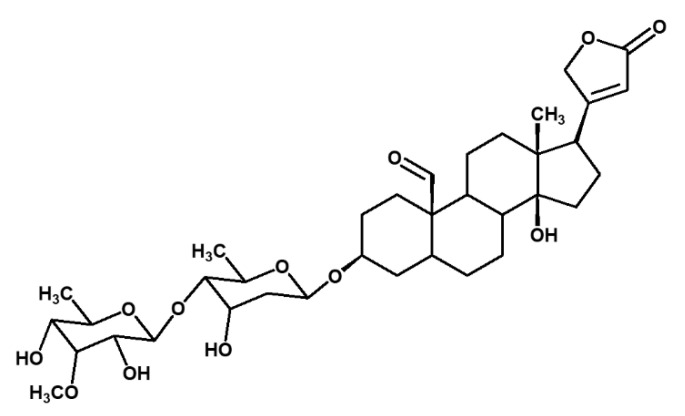
Cardenolide glycoside crotoxigenin-3-*O*-β-digitalopyranosyl-(1-4)-*O*-β-digitoxopyanoside, isolated *from X. undulatum*.

Acetylated glycosydated crotoxogenin (**1**) emerged as the most active compound in our study. The acetate group (5′-OCOCH3) attached to the sugar moiety in this compound is for the first time reported in the cardenolides, making it a novel compound. This molecule, however, is not the most potent of this class of compounds as the previously identified cardenolide crotoxigenin-3-*O*-β-digitalopyranosyl-(1-4)-*O*-β-digitoxopyanoside (Figure 8) (also isolated from *X. undulatum*) was more active, displaying a significant reduction of Aβ42 at 0.025 μM—at least 40 times more potent than any of the other cardenolides [6].

There is currently a paucity of knowledge on the efficacy of cardenolides for the treatment of AD with only one published study available from literature [6]. Nonetheless, it is encouraging to note that from the limited studies investigating the reduction of Aβ42 formation by *X. undulatum* compounds, two potent molecules have been identified. Our findings thus provide impetus for the further investigation of more plant species in search of more AD treatment compounds.

However, of concern regarding cardenolides is their well-described cardiac toxicity [18]. This potentially could retard their development into clinically approved drugs. Since the butenolide ring in cardenolides has been identified as being responsible for the cardiotoxicity of this class of compounds [19], it may be worthwhile to investigate glycoside derivatives lacking the butenolide ring. Should these derivatives retain potency, they could serve as better chemical scaffolds to develop new lead compounds. This comes as glycosides are already extensively used in the clinical treatment of bacterial infections, with a good safety record making them a pharmaceutically attractive scaffold to work on [20]. More enthusiasm on glycosides can be drawn from the evidence that they could be pluripotent AD treatment agents; they have been reported to additionally inhibit acetylcholinesterase enzymes, which are well-established and characterized AD druggable targets [21]. Glycosides have the added advantage of being able to penetrate the blood–brain barrier (BBB) [22,23] (>98% of small molecules are not able to penetrate the BBB), a major stumbling block which retards development of AD therapeutics [24]. In fact, in some studies it has been demonstrated that glycosylation of some compounds has significantly improved their BBB penetration [25,26].

Preliminary data from our docking studies suggest that the glycoside moiety on cardenolides plays a more prominent role in the binding of the molecule to its predicted biological target, BACE1 (a proteolytic enzyme hypothesized to initiate Aβ formation by cleavage of APP’s [27]), with additional role attributed to the steroid ring and acetyl group of the glycoside **1**, while the butenolide seems to play a less significant role. Similarly, glucose units on flavanone glycosides have been shown to play a significant role in the strengthening of the protein–ligand complex via hydrogen bonding and hydrophobic interactions [21]. Interestingly, flavanones with two glucose units have been reported to be more potent against BACE1 compared with those with either one glucose moiety or none [21]. This is in agreement with data from our studies which have shown crotoxigenin-3-O-β-digitalopyranosyl-(1-4)-*O*-β-digitoxopyanoside, which has two sugar moieties, to be the most potent while those with one glucose unit, namely compounds **1** and **2**, were comparatively less potent [6].

## 4. Materials and Methods

### 4.1. Plant Extraction, Fractionation, and Isolation of Compounds

Extraction and fractionation steps were meticulously carried out in consistency with our previous study [6]. Fresh leaves of *X. undulatum* were collected from the experimental farm at the University of Pretoria. A voucher specimen (PRU 124301) was prepared and deposited at the H.G.W.J. Schweickerdt Herbarium at the University of Pretoria. Oven-dried leaves of *X. undulatum* were extracted using dichloromethane and methanol (DCM:MeOH) (1:1) [6]. The resulting crude extract was subjected to silica gel column chromatography with a stepwise sequential gradient of DCM: hexane (50:50–80:20), DCM (100%), DCM: methanol (95:05–70:30) for elution resulting in the collection of fractions which were then pooled together following thin layer chromatography phytochemical profiling. Fractions 14 and 15 were combined (5.2 g).

Preparative high-performance liquid chromatography–mass spectrometery (prep HPLC-MS) on a Waters chromatographic system equipped with Waters photodiode array (PDA) detector (Model 2998) and a QDa mass spectrometer (Waters, Milford, MA, USA) was used to purify the active fractions. The separation was achieved on an XBridge preparative C18 column (19 mm × 25 mm, i.d., 5 μm particle size, Waters). The mobile phase was 0.1% formic acid in water (solvent A) and acetonitrile (solvent B). The flow rate of the mobile phase was 20 mL/min and injection volume was 150 µL. The gradient elution was as follows: the initial ratio was 5% solvent B maintained for 1 min, increasing to 50% solvent B (1:00–8:00 min), then increasing from 50% to 85% solvent B (8:00–13:00 min), and finally returned to the initial ratio of 5% solvent B within 1:20 min (13:00–13:20 min). The pure compounds were collected using a fraction collector and subsequently combined and concentrated using the speed vacuum.

### 4.2. Aβ Peptide Assay

To assess the ability of isolated compounds to block the formation of Aβ-proteins from APP’s, the Aβ assay was carried out as previously described [6]. The samples were prepared by dissolving the stock solution of the compound in DMSO and kept at −20 °C. Before each experiment, the solution was diluted to the final concentration in fresh media. In order not to affect cell growth, the final DMSO concentration did not exceed 0.5% in all experiments.

APPsw-transfected HeLa cells at 80% confluence in a 35 mm dish were cultured for 8 h with purified compound solubilized in dimethyl sulphoxide (DMSO) in a medium without serum. As per supplier’s instruction, the conditioned medium was analysed by using enzyme-linked immunosorbent assay (ELISA; Invitrogen, CA, USA) to detect Aβ42, Aβ40, and sAPPβ-sw [6]. Data were collected using 2 replicated experiments; 2 dishes are used for each independent experiment.

### 4.3. MS and NMR Analysis

Isolated compounds were analysed by UPLC-QTOF-MS/MS using a Waters Acquity™ UPLC instrument coupled to a Waters Synapt G2 high definition MS as previously described [6]. The data acquisition was done by using the software MassLynx 4.19 (Waters Inc., Milford, Massachusetts, MA, USA). The sample (1 mg/mL) was prepared by dissolving dried extract, fraction, or pure compounds in 100% methanol. The solution was centrifuged at 10,000× *g* for 10 min to remove particulates. The analysis was done using Waters BEH C18 1.7 µm particle size (2.1 mm × 100 mm) column. The injected volume was 5 µL and the elution flow rate was 0.3 mL/min. The mobile phases were A, water + 0.1% formic acid and B, methanol + 0.1% formic acid. The gradient elution used was 0% to 3% B, 0 min to 0.1 min; 3% to 100% B, 0.3 min to 14.00 min; 100% B, 14.00 min to 16.00 min, 100% to 3% B, 16.00 to 16.50; and 3% B, 16.50 to 20.00 min. Using 1D (^1^H, ^13^C and ^13^C DEPT-135) and 2D (HSQC, HMBC, and COSY) NMR spectroscopy, the structure elucidation of the pure compounds was completed. The NMR spectra were recorded on a 400 MHz Bruker Advance III spectrometer at 25 °C. The isolated compounds were dissolved in deuterated methanol (Aldrich Chemistry, Sigma-Aldrich, Milwaukee, Wisconsin, WI, USA) with chemical shifts of isolated compounds referenced to it (MeOD-*d4*, δH 3.31; δC 49.0 ppm).

### 4.4. Target and Ligand Preparation

Schrödinger Release 2019-2 software suite was utilized for all in silico modelling. Selected crystal structures for *BACE1* were retrieved from the PDB database. The complexes were prepared with the *protein preparation wizard* found within *Maestro* [28]. The preparation was done to fill in the missing side chain or loops, assign the correct bond orders, optimize the hydrogen bonding network, and minimize the system to alleviate any mild clashes. Complexes were superimposed on each other using the Cα backbone atoms of the protein. The binding pockets of the complexes were visually inspected to identify unique conformations of BACE1. The following crystal structures were selected for docking: 1XS7, 2QZK, 2F3E, 2F3F, 3DV1, 3DV5, 3K5C, 4K8S, and 4KE0. Receptor grids of the selected complexes were generated using the *receptor grid generation* tool within *Maestro*. Water molecules within the binding pocket which formed several hydrogen bonds with residues of the surrounding area were retained.

Ligands **1**–**3** were prepared with LigPrep [29] using the default settings. However, enumeration of the stereocenters was not performed because the stereochemistry for the ligands were known.

#### 4.4.1. Molecular Docking

Molecular docking was performed with *Glide* extra-precision (XP) [30,31,32,33]. *Glide* is known to produce superior predictions compared with other docking programs based on benchmark studies [32,33]. Ligand structures were treated as flexible, which allowed for sampling of ring conformations. Default settings for the program were utilized.

#### 4.4.2. Induced Fit Docking

The ligands were also submitted for Induced Fit Docking (IFD) [34,35] from the Schrödinger software suite in which an algorithm attempts to find the optimal binding pose of the ligand if the residues in the binding pocket are considered flexible. Solvent molecules within the binding pocket from the docking procedure were kept for the IFD. The box centre was obtained using residues Gln73, Lys107, Trp115, Asp228, Thr232 and Arg307. Apart from the receptor van der Waals scaling, the default settings were used, which was reduced to 0.40.

### 4.5. Statistical Analysis

Data were analysed with Prism 7.0 software (GraphPad Software, Inc., San Diego, CA, USA) using one-way analysis of variance (ANOVA) followed by the Tukey multiple comparison test.

## 5. Conclusions

The bioassay-guided approach utilized in the study led to the isolation of the active ingredients in *X. undulatum* responsible for the reduction of Aβ42 level in vitro. Three cardenolide glycosides, acetylated glycosydated crotoxigenin **1**, xysmalogenin-3, β-d-glucopyranoside **2**, and crotoxigenin 3-*O*-glucopyranoside **3,** were identified using spectral techniques. Acetylated glycosydated crotoxigenin is reported as a novel compound, and the detailed 2D NMR data for xysmalogenin-3, β-d-glucopyranoside, and crotoxigenin 3-*O*-glucopyranoside has been published for the first time in this study. Often the approach of bioassay-guided fraction is a challenge to natural product chemists as bioactivity is lost during fractionation for various reasons, however, in this case, potency of the fractions and the isolated compounds increased during the purification process. Of these three compounds, acetylated glycosydated crotoxigenin and xysmalogenin-3, β-d-glucopyranoside showed for the first-time strong activity towards the reduction of Aβ42. Preliminary computational studies provided a tentative binding pose of compound **1** in BACE1, however, this will need to be followed up with a more extensive computational analysis which uses long molecular dynamic simulations and binding pose metadynamics [36] to confirm the binding pose and explain the inactivity of **3**. Future work will involve the long molecular dynamic simulation, removal of the butenolide ring, and testing the glycosydated aglycones for their potential to reduce Aβ42. This will aid in the anticipated medicinal chemistry studies of these compounds as part of work to develop them into lead compounds.

## Figures and Tables

**Table 1:** ^1^H NMR and ^13^C NMR data for glycosydated crotoxigenin (**1**) in MeOD-*d4*.

Position	^13^C (ppm)	^1^H (ppm), *J* (Hz)	COSY	HMBC
1a	28.1	1.62, m		C-9
1b		1.51, m		
2a	33.0	1.96, m		C-3, C-10
2b		1.78, m		
3	77.8	3.74, m		
4a	25.9	1.87, m		
4b		1.37, m		
5	41.6	1.89, m		C-9
6a	25.1	1.92, m		C-5
6b		1.35, m		
7a	21.2	1.80, m		C-6, C-8
7b		1.34, m		
8	35.0	1.98, m		C-5
9	35.1	1.83, m		
10	50.5	-		
11a	20.3	1.79, m		C-9, C-10
11b		1.51, m		
12	39.3	1.88, m		C-9
12b		1.57, m		
13	49.6	-		
14	84.5	-		
15a	31.3	2.21, m		
15b		1.72, m		
16a	26.5	2.17, m		C-14
16b		1.61, m		
17	50.4	2.80 (q, 14.6)		C-13, C-14, C-21, C-22
18	14.8	0.97, s		C-12, C-13, C-14
19	206.5	9.57, s		C-9
20	175.8	-		
21a	73.9	5.03 (dd, 18.5, 1.57)		C-22
21b		4.96 (dd, 18.1, 1.73)		
22	116.4	5.92, s		C-17, C-21, C-23
23	176.9	-		
1′	101.3	4.40 (d, 7.57)	H-2′	C-3
2′	73.6	3.17, m	H-1′, H-3′	C-1′
3′	70.2	3.36, m	H-2′	
4′	73.7	3.30, m	H-5′	
5′	76.4	3.47, m	H-4′	
6′a	63.4	4.20 (dd, 11.6, 5.14)	H-5′	C-2, C-5, -OCOCH_3_
6′b		4.30 (dd, 11.6, 1.84)		
5′- OCO	171.2	-		
5′- CH_3_	19.3	2.08, s		C-5′-OCO

**Table 2:** ^1^H NMR and ^13^C NMR data for xysmalogenin-3, β-d-glucopyranoside (**2**) in MeOD-*d4*.

Position	^13^C (ppm)	^1^H (ppm), *J* (Hz)	COSY	HMBC
1	38.9	2.27, m		C-3, C-5, C-6
2a	30.5	1.92, m		
2b		1.62, m		
3	79.6	3.60, m		
4	40.1	1.52, m		
5	140.8	-		
6	122.7	5.44, m	H-7	C-8
7a	27.4	2.26, m	H-6	C-5, C-6
7b		2.22, m		
8	38.3	1.72, m		
9	47.7	1.24, m		
10	38.3			
11	22.0	1.53, m		
12	39.4	2.44, m		
13	50.7	-		
14	86.3	-		
15	33.8	1.74, m		
16	28.1	1.73, m		C-14, C-15
17	52.1	2.86, (q, 14.62)		C-12, C-21, C-22, C-23
18	16.1	0.92, s		C-12, C-13, C-14
19	19.8	1.03, s		C-5, C-9, C-10
20	177.1	-		
21a	75.2	5.02, (dd, 18.44, 1.65)		C-22, C-23
21b		4.92, (dd, 18.44, 1.65)		
22	117.8	5.91, s		C-13, C-20, C-21
23	178.2	-		
1′	102.3	4.38 (dd, 7.68)	H-2′	C-3
2′	75.1	3.14, m		C-1′, C-3′
3′	78.0	3.25, m		
4′	71.5	3.25, m		
5′	77.7	3.35, m		
6′a	63.6	3.84, m	H-5′	C-4′, C-5,
6′b		3.65, m		

## Data Availability

Data is contained within the article and supplementary material.

## Supplementary Materials

The following are available online at https://www.mdpi.com/article/10.3390/ph14080743/s1, Figure S1: iFit value of acetylated glycosydated crotoxigenin **1** in *X. undulatum* leaves extracted with DCM:MeOH, Figure S2: ^1^H NMR for acetylated glycosydated crotoxigenin **1** in MeOD-*d4*, Figure S3: ^13^C NMR for acetylated glycosydated crotoxigenin **1** in MeOD-*d4*, Figure S4: DEPT 135 for acetylated glycosydated crotoxigenin **1** in MeOD-*d4*, Figure S5: HSQC spectrum for acetylated glycosydated crotoxogenin **1** in MeOD-*d4*, Figure S6: COSY spectrum for acetylated glycosydated crotoxogenin **1** in MeOD-*d4*, Figure S7: HMBC for acetylated glycosydated crotoxigenin **1** in MeOD-*d4*, Figure S8: iFit value of Xysmalogenin-3, β-d-glucopyranoside **2** in *X. undulatum* leaves extracted with DCM:MeOH, Figure S9: ^1^H NMR for Xysmalogenin-3, β-d-glucopyranoside **2** in MeOD-*d4*, Figure S10: ^13^C NMR for Xysmalogenin-3, β-d-glucopyranoside **2** in MeOD-*d4*, Figure S11: DEPT 135 for Xysmalogenin-3, β-d-glucopyranoside **2** in MeOD-*d4*, Figure S12: HSQC for Xysmalogenin-3, β-d-glucopyranoside **2** in MeOD-*d4*, Figure S13: COSY spectrum for Xysmalogenin-3, β-d-glucopyranoside **2** in MeOD-*d4*, Figure S14: HMBC spectrum for Xysmalogenin-3, β-d-glucopyranoside **2** in MeOD-*d4*, Figure S15: iFit value of crotoxigenin 3-*O*-glucopyranoside **3** in *X. undulatum* leaves extracted with DCM:MeOH, Figure S16: (**a**) Predicted binding pose from induced fit docking for **2**, shown in green, bound to BACE1. Hydrogen bonds are shown in yellow dashed lines. Only selected residues are shown for clarity. (**b**) 2D ligand interaction diagram for the binding pose of **2**, Figure S17: (**a**) Predicted binding pose from induced fit docking for **3**, shown in green, bound to BACE1. Hydrogen bonds are shown in yellow dashed lines. Only selected residues are shown for clarity. (**b**) 2D ligand interaction diagram for the binding pose of **3**, Table S1: ^1^H NMR and ^13^C NMR data for crotoxogenin 3-*O*-glucopyranoside **3** in MeOD-*d4* compared with literature reports for the compound in DMSO-*d6*.
