# Supplementary material for: A Novel Cardenolide Glycoside Isolated from Xysmalobium undulatum Reduces Levels of the Alzheimer’s Disease-Associated β-Amyloid Peptides Aβ42 In Vitro
Source: Pharmaceuticals (Basel). 2021 Jul 29;14(8):743. doi: 10.3390/ph14080743 (PMC8400651; doi:10.3390/ph14080743)
Supplement: Supplementary file 1 [file pharmaceuticals-14-00743-s001.zip › pharmaceuticals-1270210-supplementary.pdf]

Supplementary Materials:

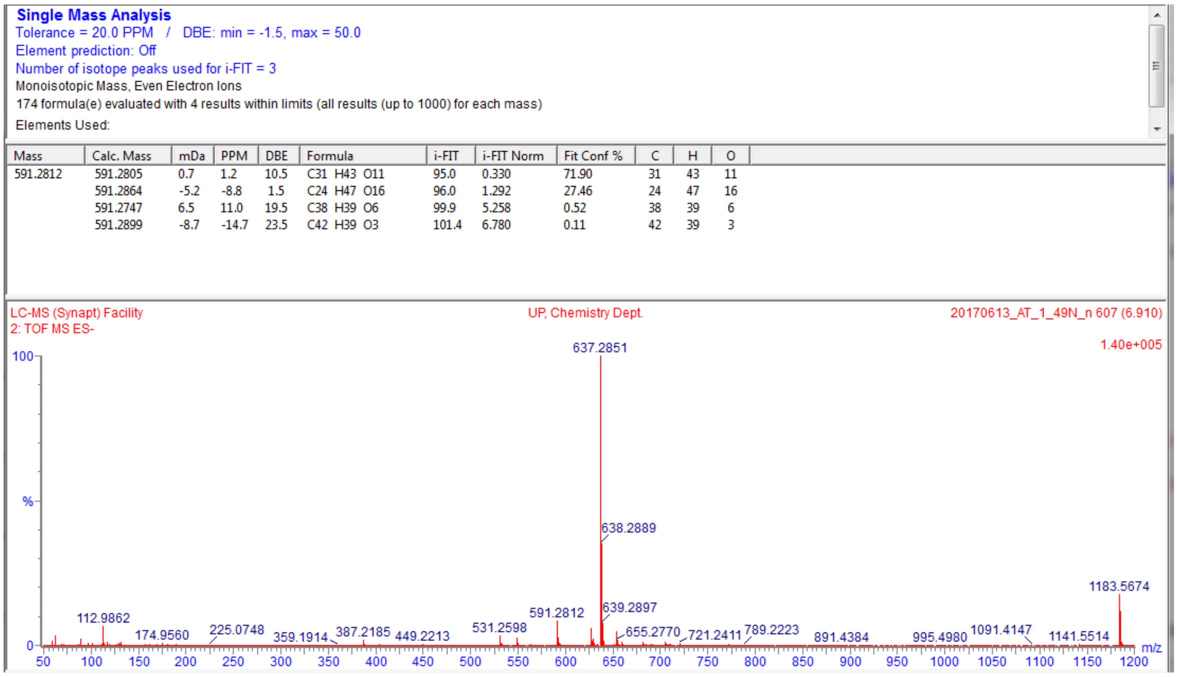

Supplementary Figure S1. iFit value of acetylated glycosydated crotoxigenin **1** in *X. undulatum* leaves extracted with DCM:MeOH

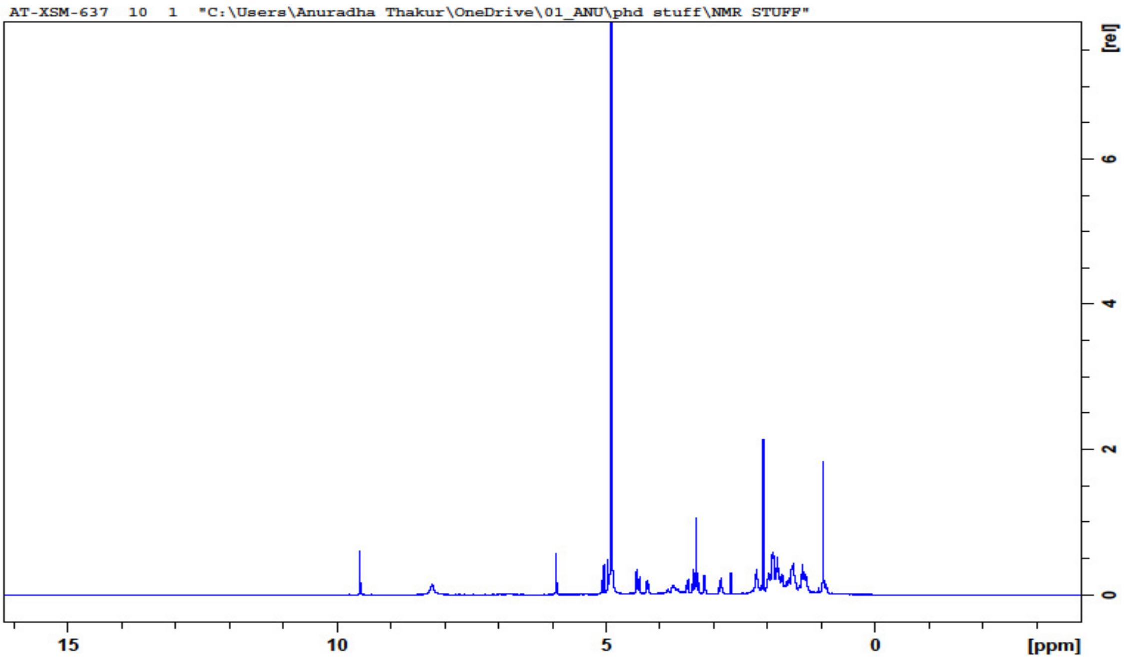

Supplementary Figure S2. ^{1}H NMR for acetylated glycosydated crotoxigenin **1** in MeOD-*d4*

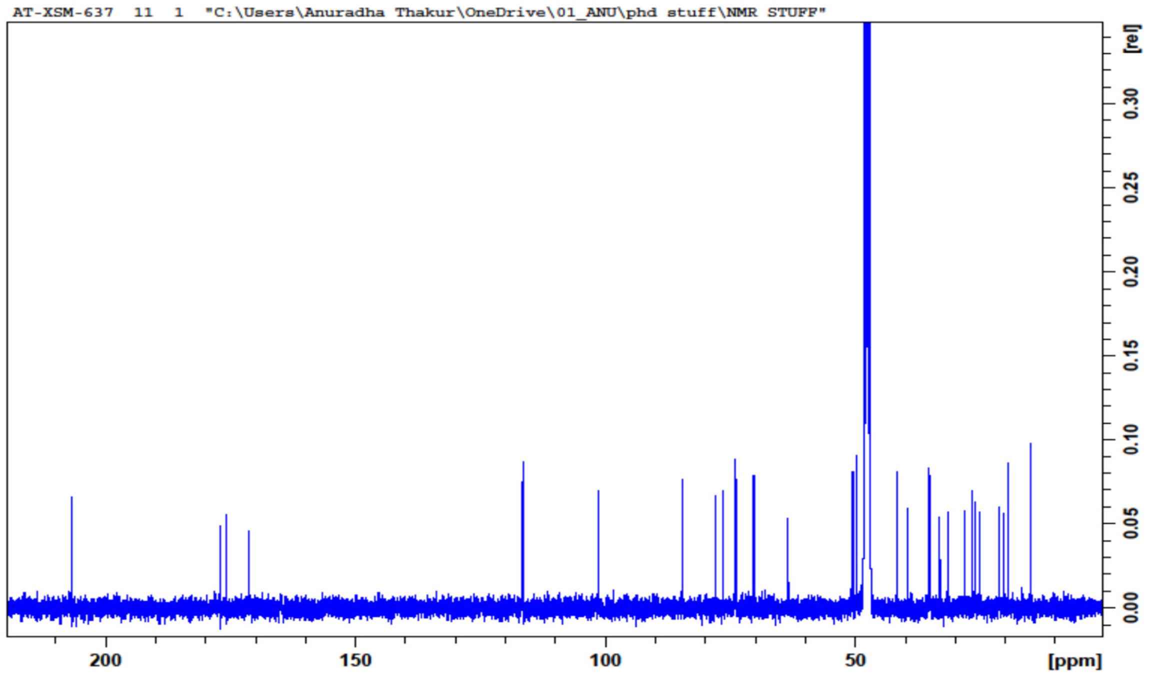

Supplementary Figure S3. ^{13}C NMR for acetylated glycosydated crotoxigenin **1** in MeOD-*d4*

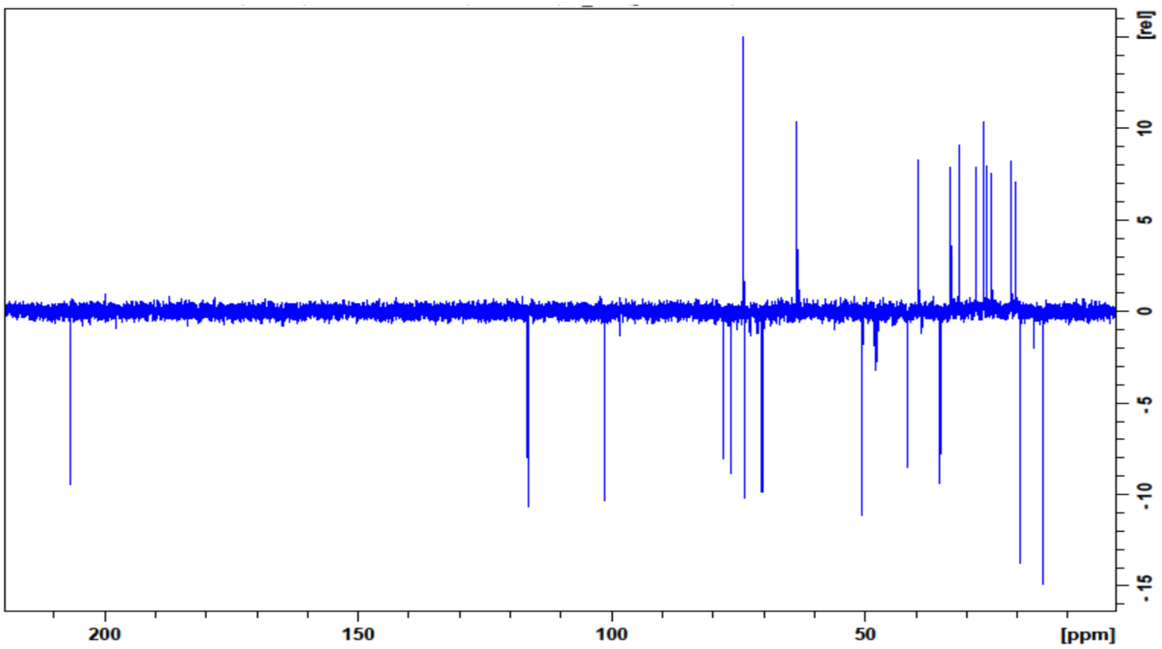

Supplementary Figure S4. DEPT 135 for acetylated glycosydated crotoxigenin **1** in MeOD-*d4*

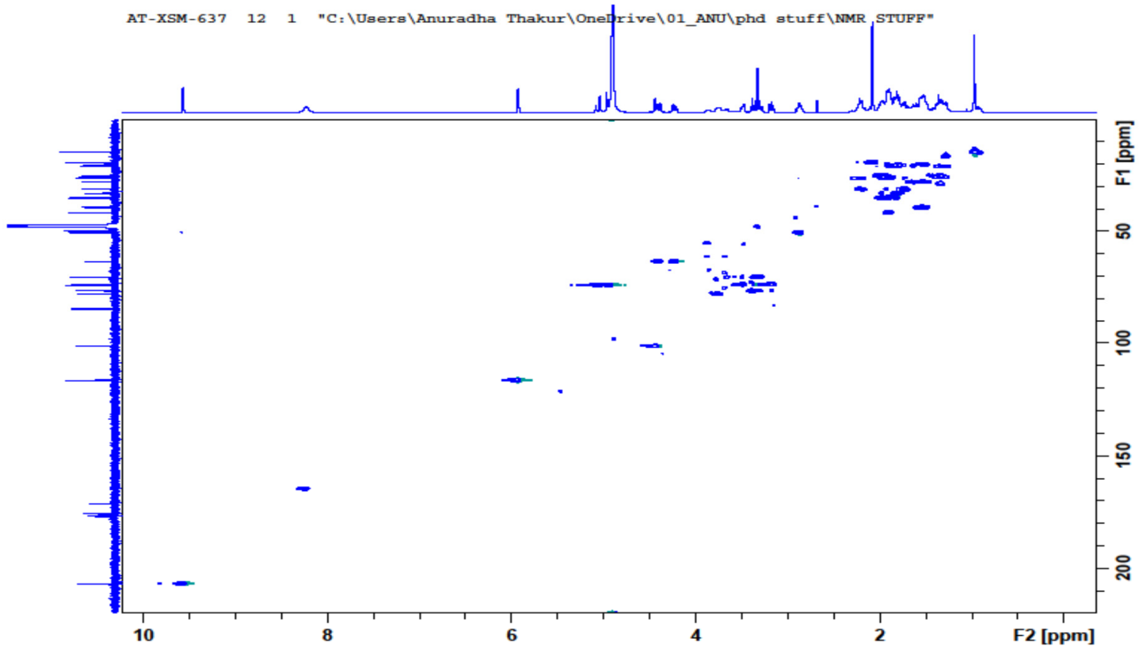

Supplementary Figure S5. HSQC spectrum for acetylated glycosydated crotoxogenin **1** in MeOD-*d4*

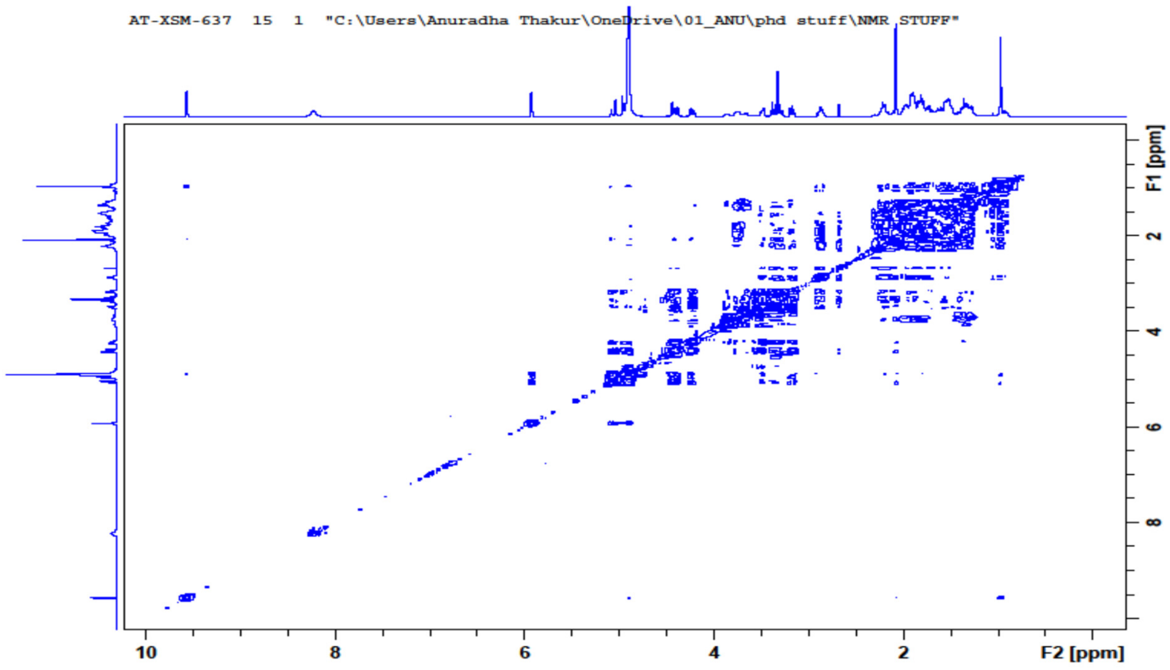

Supplementary Figure S6. COSY spectrum for acetylated glycosydated crotoxogenin **1** in MeOD-*d4*

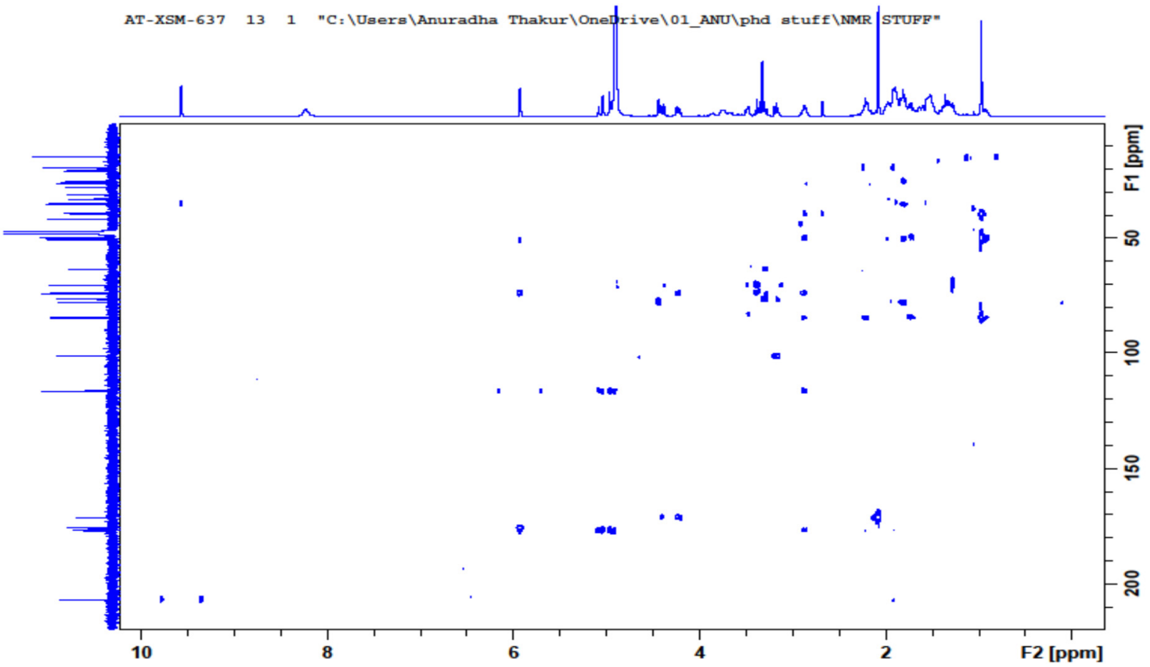

Supplementary Figure S7. HMBC for acetylated glycosydated crotoxigenin **1** in MeOD-*d4*

Single Mass Analysis
Tolerance = 20.0 PPM / DBE: min = -1.5, max = 50.0
Element prediction: Off
Number of isotope peaks used for i-FIT = 3
Monoisotopic Mass, Even Electron Ions
141 formula(e) evaluated with 4 results within limits (all results (up to 1000) for each mass)
Elements Used:

Mass	Calc. Mass	mDa	PPM	DBE	Formula	i-FIT	i-FIT Norm	Fit Conf %	C	H	O
533.2756	533.2751	0.5	0.9	9.5	C29 H41 O9	286.5	0.001	99.94	29	41	9
	533.2809	-5.3	-9.9	0.5	C22 H45 O14	294.0	7.476	0.06	22	45	14
	533.2692	6.4	12.0	18.5	C36 H37 O4	296.5	9.998	0.00	36	37	4
	533.2844	-8.8	-16.5	22.5	C40 H37 O	299.0	12.519	0.00	40	37	1

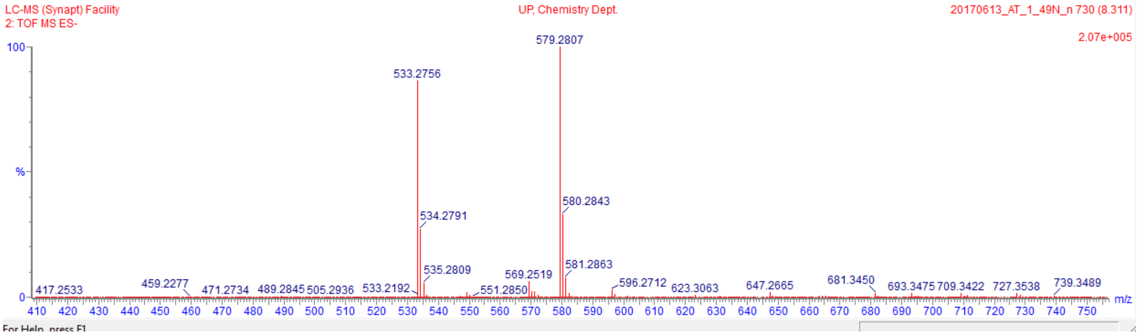

Supplementary Figure S8. iFit value of Xysmalogenin-3, β-D-glucopyranoside **2** in *X. undulatum* leaves extracted with DCM:MeOH

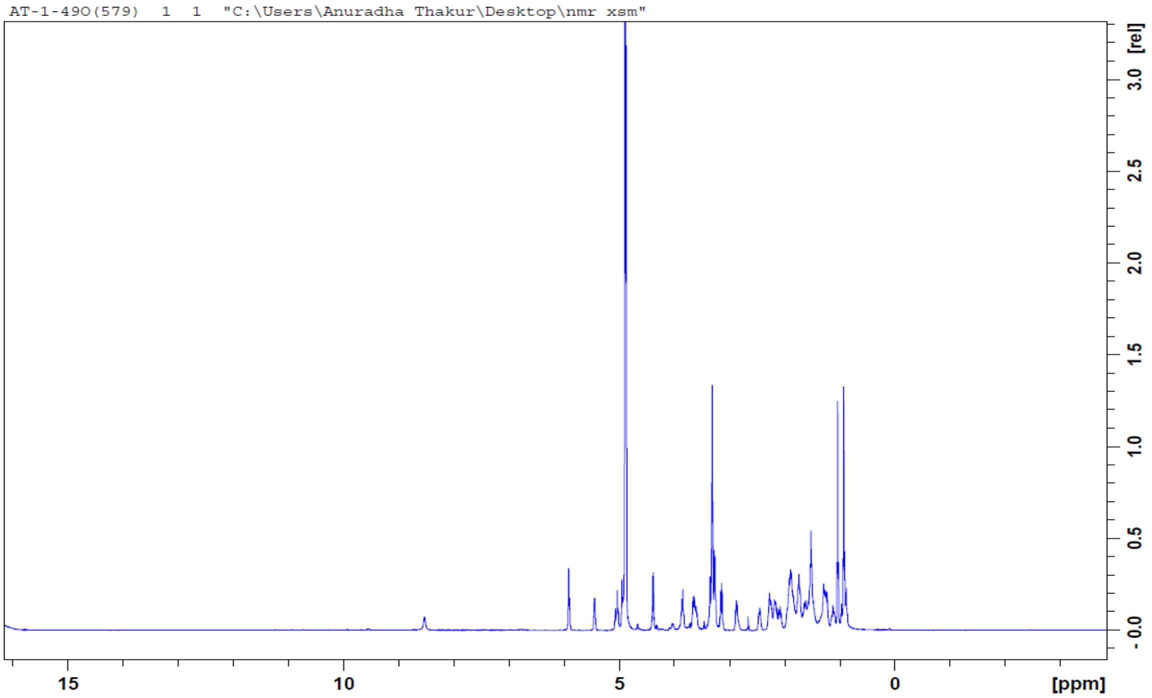

Supplementary Figure S9. ^{1}H NMR for Xysmalogenin-3, β-D-glucopyranoside **2** in MeOD-*d4*

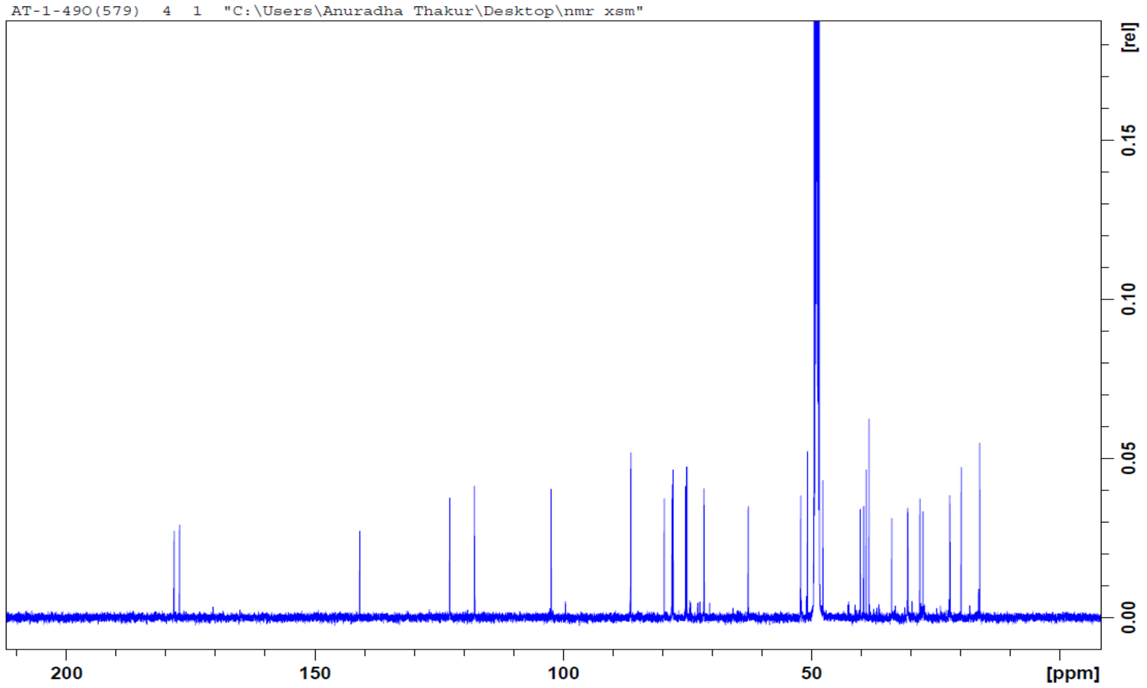

Supplementary Figure S10. ^{13}C NMR for Xysmalogenin-3, β-D-glucopyranoside **2** in MeOD-*d4*

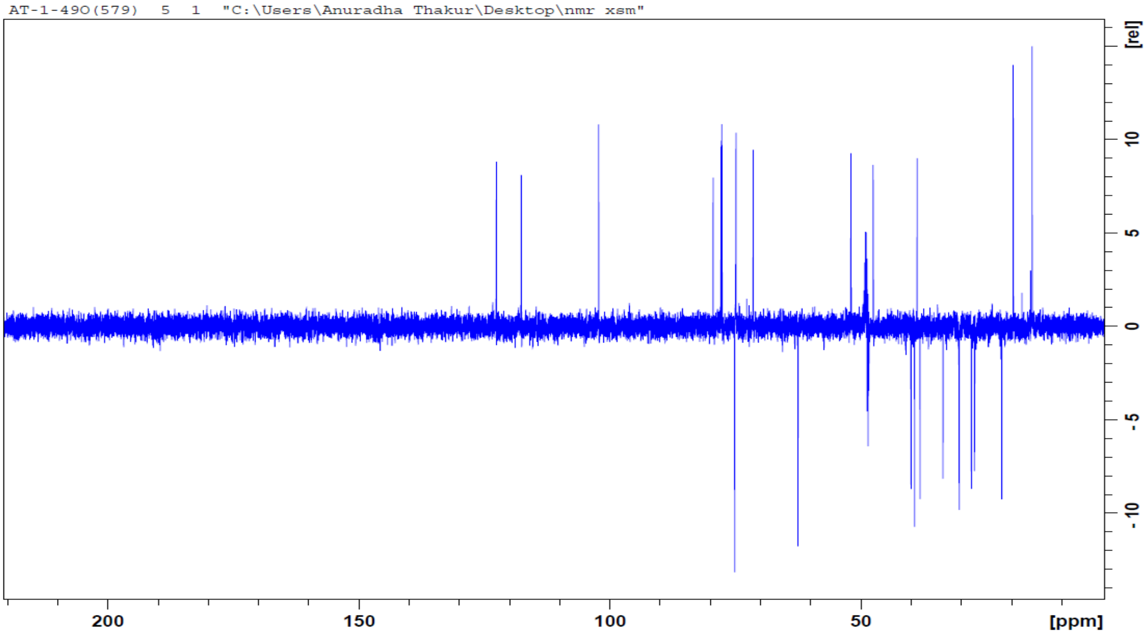

Supplementary Figure S11. DEPT 135 for Xysmalogenin-3, β-D-glucopyranoside **2** in MeOD-*d4*

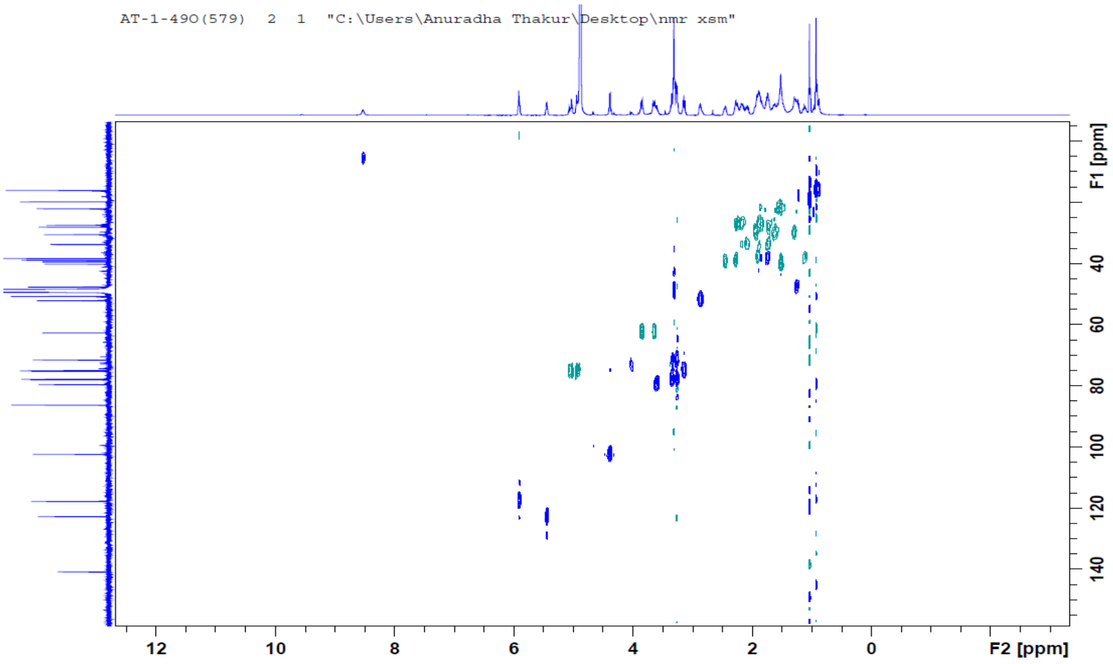

Supplementary Figure S12. HSQC for Xysmalogenin-3, β-D-glucopyranoside **2** in MeOD-*d4*

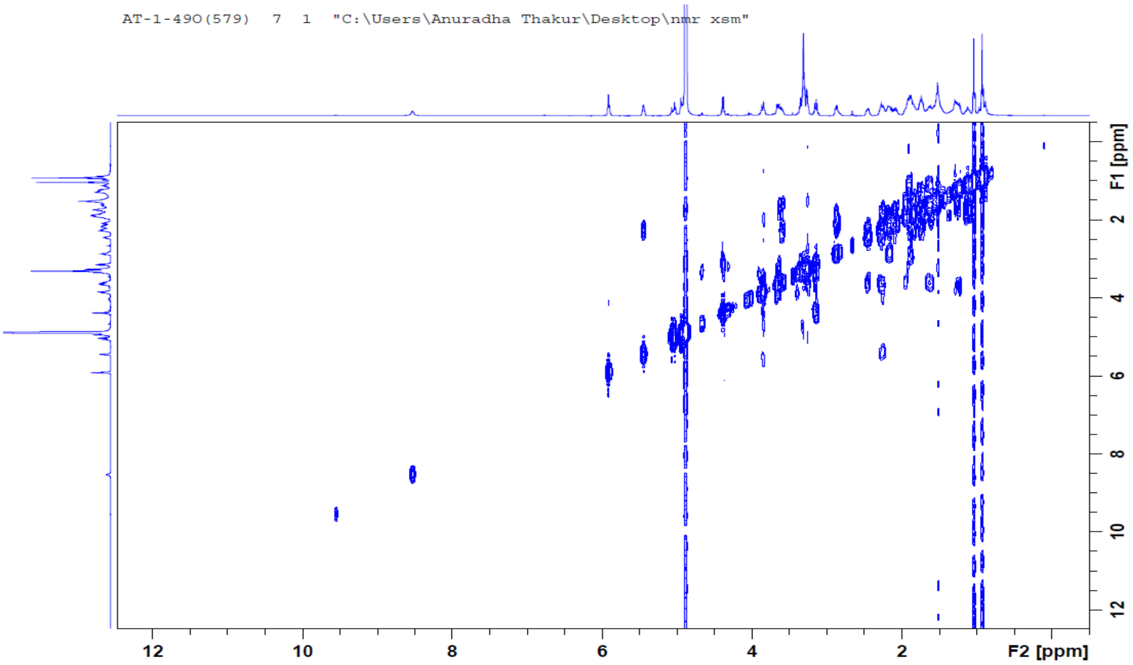

Supplementary Figure S13. COSY spectrum for Xysmalogenin-3, β-D-glucopyranoside **2** in MeOD-*d4*

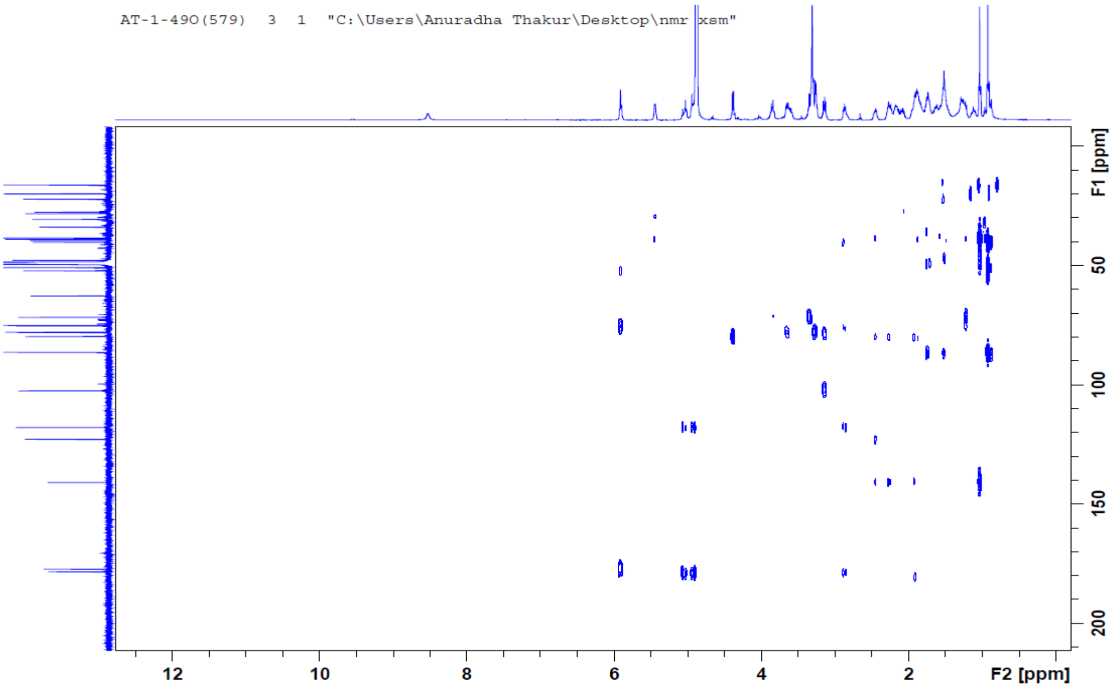

Supplementary Figure S14. HMBC spectrum for Xysmalogenin-3, β-D-glucopyranoside **2** in MeOD-*d4*

Single Mass Analysis
Tolerance = 20.0 PPM / DBE: min = -1.5, max = 50.0
Element prediction: Off
Number of isotope peaks used for i-FIT = 3
Monoisotopic Mass, Even Electron Ions
148 formula(e) evaluated with 4 results within limits (all results (up to 1000) for each mass)
Elements Used:

Mass	Calc. Mass	mDa	PPM	DBE	Formula	i-FIT	i-FIT Norm	Fit Conf %	C	H	O
549.2707	549.2700	0.7	1.3	9.5	C29 H41 O10	218.4	0.005	99.46	29	41	10
	549.2758	-5.1	-9.3	0.5	C22 H45 O15	224.3	5.905	0.27	22	45	15
	549.2641	6.6	12.0	18.5	C36 H37 O5	224.5	6.028	0.24	36	37	5
	549.2794	-8.7	-15.8	22.5	C40 H37 O2	226.8	8.355	0.02	40	37	2

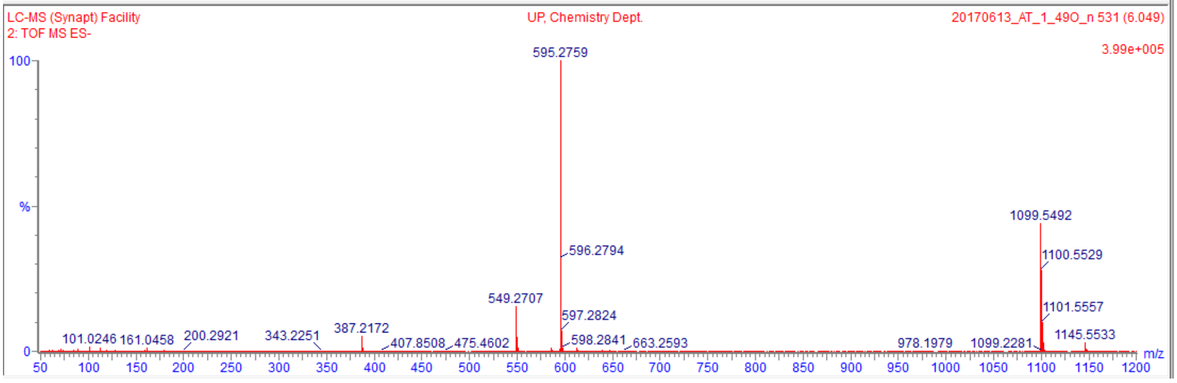

Supplementary Figure S15. iFit value of crotoxigenin 3-O-glucopyranoside **3** in *X. undulatum* leaves extracted with DCM:MeOH

Supplementary Table S1. ^{1}H NMR and ^{13}C NMR data for crotoxogenin 3-*O*-glucopyranoside **3** in MeOD-*d4* compared to literature reports [1] for the compound in DMSO-*d6*.

Isolated Crotoxogenin 3-O-glucopyranoside (18)			^{1}H NMR and ^{13}C NMR literature data for compound (59) in DMSO-d6	
**Position**	**^{13}C (ppm)**	**^{1}H (ppm), *J* (Hz)**	**^{13}C (ppm)**	**^{1}H (ppm), *J* (Hz)**
1	28.0	1.83 m, 1.54 m	28.03	1.85 m,1.50 m
2	31.3	1.85 m	30.36	1.80 m
3	77.0	3.82 m	76.78	3.05 m
4	25.9	1.85 m, 1.33 m	30.20	1.82 m, 1.14 m
5	41.6	1.89 m	42.03	1.45 m
6	24.9	1.76 m	26.27	1.74 m
7	20.3	1.48 m	21.41	1.60 m
8	35.2	1.96 m	47.57	1.43 m
9	35.0	1.79 m	35.49	1.81 m
10	50.4	-	49.99	-
11	21.2	1.82 m, 1.32 m	21.41	1.60 m
12	39.4	1.51 m	42.18	1.44 m
13	49.6	-	49.19	-
14	84.5	-	83.37	-
15	32.9	1.71 m	31.59	1.94 m, 1.55 m
16	26.5	2.16 m	27.17	2.13 m
17	50.5	2.8 (q, 14.6)	51.23	2.72 m
18	14.8	0.95 s	15.54	0.69 s
19	206.8	9.54 s	209.66	9.94 s
20	175.8	-	173.96	-
21	73.7	5.04 (d, 1.65), 4.92 (dd, 18.5, 1.74)	73.48	4.90 dd
22	116.4	5.91 s	116.38	5.89 s
23	176.9	-	176.31	-
1′	100.8	4.42 d (7.80)	100.71	4.20 d
2′	73.9	3.14 (dd, 9.03, 1.07)	75.75	3.59 m
3′	76.5	3.26	73.23	2.85 m
4′	70.3	3.27 m	70.11	3.02 m
5′	76.7	3.35m	76.78	3.06 m
6′	61.4	3.87 m, 3.65 m	61.14	3.64m, 3.40m

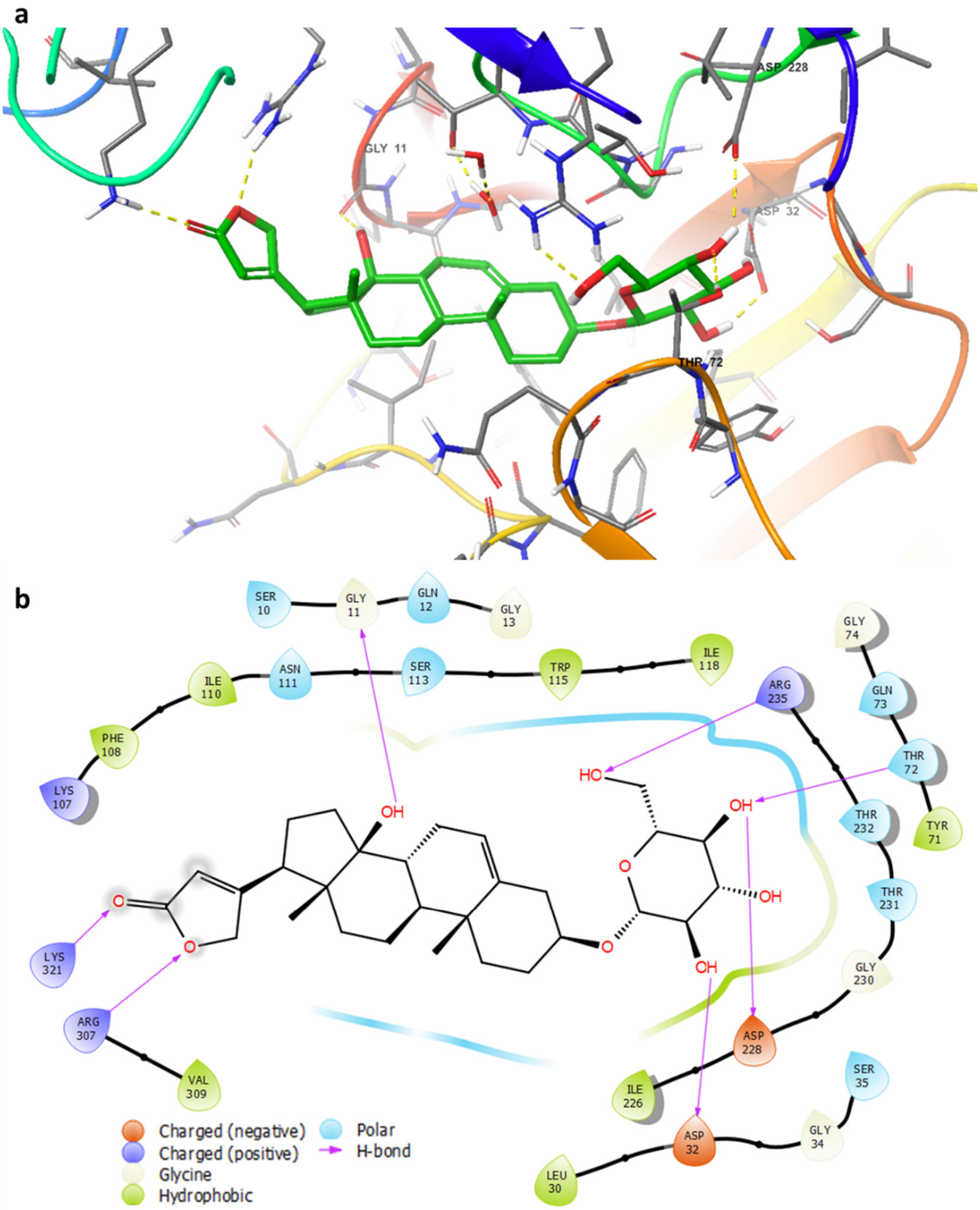

Supplementary Figure S16. (a) Predicted binding pose from induced fit docking for **2**, shown in green, bound to BACE1. Hydrogen bonds are shown in yellow dashed lines. Only selected residues are shown for clarity. (b) 2D ligand interaction diagram for the binding pose of **2**.

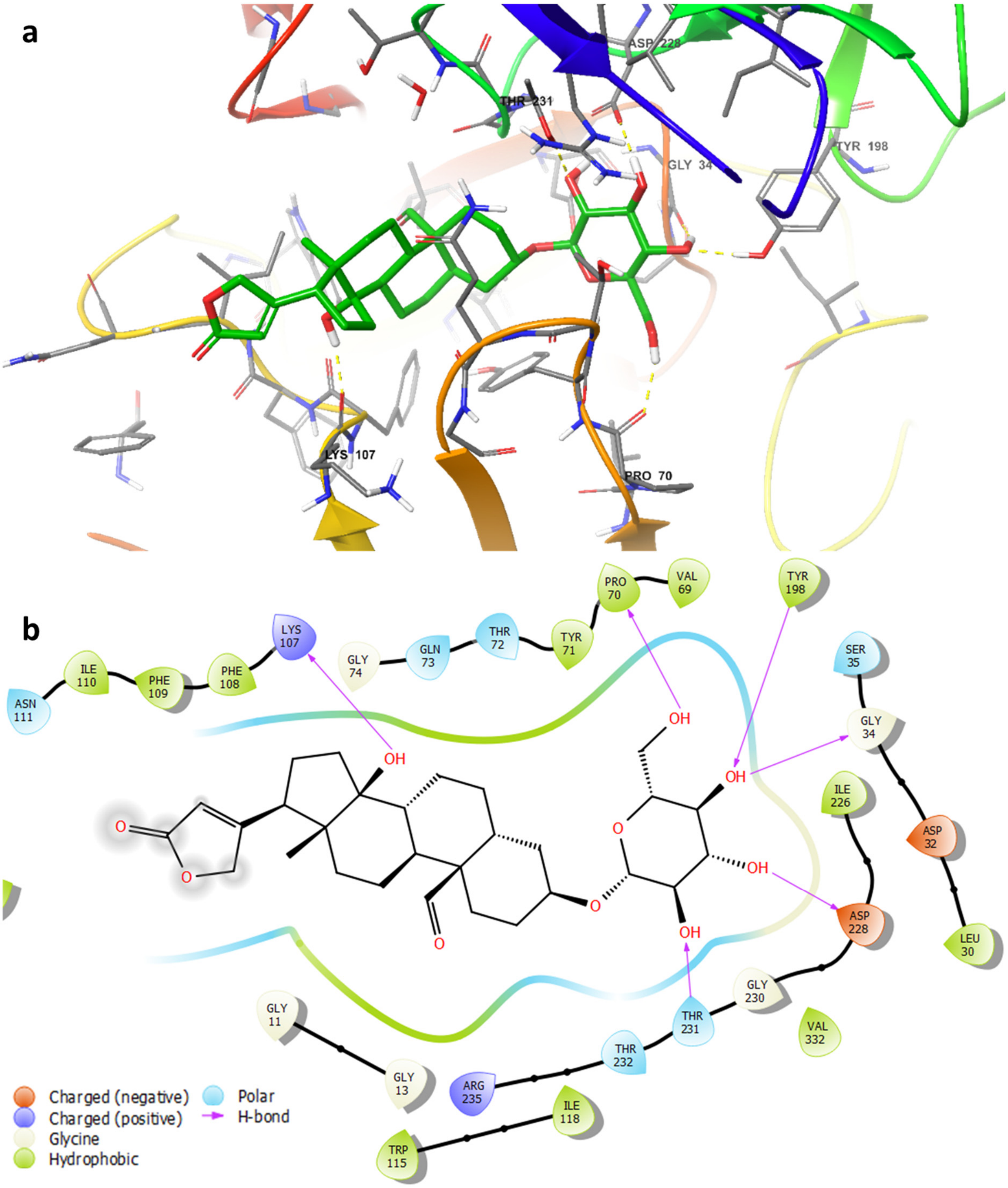

Supplementary Figure S17. (a) Predicted binding pose from induced fit docking for **3**, shown in green, bound to BACE1. Hydrogen bonds are shown in yellow dashed lines. Only selected residues are shown for clarity. (b) 2D ligand interaction diagram for the binding pose of **3**.

References:

[1] L. Rascón-Valenzuela, C. Velázquez, A. Garibay-Escobar, L. A. Medina-Juárez, W. Vilegas, and R. E. Robles-Zepeda, “Antiproliferative activity of cardenolide glycosides from Asclepias subulata,” *J. Ethnopharmacol.*, vol. 171, pp. 280–286, 2015, doi: 10.1016/j.jep.2015.05.057.
